# Control of Fluoride Pollution in Cemented Phosphogypsum Backfill by Citric Acid Pretreatment

**DOI:** 10.3390/ma16196493

**Published:** 2023-09-29

**Authors:** Yanan Zhou, Ying Shi, Quanqi Zhu

**Affiliations:** School of Resources and Safety Engineering, Central South University, Changsha 410083, China

**Keywords:** phosphogypsum, cemented backfill, fluoride, citric acid washing, environmental behavior

## Abstract

Using phosphogypsum (PG) as the aggregate of cemented backfill is an economical and effective method of PG utilization. However, the stability and performance of cemented backfill are challenged by the rich fluoride content in PG. In this study, the effects of citric acid pretreatment on PG defluorination, backfill performance and environmental behavior were investigated by washing PG with different concentrations of citric acid and washing times. The results showed that the citric acid pretreatment could significantly reduce the fluoride content in PG and promote the hydration reaction with the binder, thus greatly reducing the usage and cost of the binder in actual production. Considering the efficiency of defluorination, the optimal citric acid concentration and washing times were determined to be 4% and 7–8 times, respectively. In addition, after citric acid pretreatment, the viscosity and setting time of the backfill slurry and the porosity of the backfill reduced, and the strength of the backfill improved, which was conducive to slurry pipeline transportation and underground mine stability. Finally, a further analysis of environmental behavior was conducted and it was found that the citric acid washing greatly reduced the content of fluoride in the bleeding water of slurry and the backfill leachate, which met the integrated wastewater discharge standard in China. The results of this study can provide important guidance for the large-scale recycling and environmental management of PG.

## 1. Introduction

Phosphogypsum (PG), as a solid waste generated in the process of the phosphorus fertilizer industry (phosphoric acid production), has an annual global output of 280 million tons, and China’s annual output is about 55 million tons [1,2,3]. However, most PG is deposited without any treatment near the production sites or stockpiled at the coastal areas due to the massive generation and high transport costs [4,5]. The accumulation of waste PG in open areas will discharge various water-soluble and insoluble impurities, such as phosphates, oxides and heavy metals into the environment, which has great pollution potential for the surrounding soil, water and atmosphere and poses a threat to human health [6,7]. With the increasing requirements for environmental protection and ecological sustainability policies, PG has emerged as one of the most pressing environmental issues related to the phosphate fertilizer industry. From a sustainability perspective, recycling PG in an efficient, environmentally friendly and economical way is the core of the current solutions.

The current effective utilization of PG is less than 25% worldwide and it is mainly focused on the following initiatives: building materials, cement retarders, agricultural fertilizers and soil amendments [8,9,10]. For example, Maierdan et al. [11] used a combination of calcined PG at concentrations of 20–40% slag and cement to produce bricks with appropriate compressive strengths. Wang et al. [12] used beta-hemihydrate PG at a concentration of 50% slag, FA and alkali activator to make geopolymer cement. It should be noted that a technique using PG as an aggregate for cemented backfill in mined-out goafs has been considered to be one of the most effective and powerful initiatives for the in situ treatment of PG due to its high consumptive capacity (60–75% of PG) and economic viability [13,14]. Furthermore, the cemented backfill technique is a proven method for stabilizing/solidifying (S/S) impurities in solid waste PG. However, the high content of impurities in PG was found to hinder the implementation of the cemented PG backfill technique to some extent, especially the fluoride impurity which has a content of up to 3.6% of the mass of PG [15,16]. The presence of fluoride in PG in both soluble and insoluble forms reduces the backfill strength by inhibiting the hydration with a binder and deteriorating the backfill structure [17,18,19,20]. In addition, as the backfill slurry is transported via pipelines to the underground goaf, the fluoride that is not S/S by the binder may bleed from the backfill slurry and directly pollute the groundwater. For this reason, before using PG for cemented backfill, it is recommended that PG should be treated in some way to minimize the deleterious effects of fluoride on mine stability and the environment. 

Recently, some studies have focused on reducing and eliminating hazards caused by fluoride in PG. Bilal et al. [21] reviewed and discussed how to remove impurities such as fluoride so that PG can be used in construction. The available methods in the existing research are primarily classified into two aspects: immobilization and removal. One of the most commonly used immobilization methods is to pre-add alkaline additives containing Ca^2+^ to PG before secondary utilization, converting soluble fluoride to insoluble Ca-F compounds, thereby improving the quality of PG [1,22]. However, the solubility of Ca-F compounds will result in a strong alkaline condition, resulting in inadequate control of fluoride leaching when using the pretreated PG to make backfill [23]. Some methods for fluoride removal, including liquid washing, have also been applied to PG. However, it was found that the water-washing pretreatment was effective in removing soluble fluoride, whereas the insoluble fluoride was challenging to be washed out [24]. Although the insoluble fluoride present in PG could be eliminated by washing with H_2_SO_4_, the crystal structure of PG was shown to be altered [25]. Fortunately, a fluoride removal method of washing PG with citric acid has been proven to be effective. Singh [26] washed PG with citric acid, which removed both soluble and insoluble fluoride in the form of water-soluble citrate, aluminates and ferrates, resulting in a reduction in fluorine content from 1.5% to 0.65%. Additionally, previous studies have shown that the strength of hardened structures prepared from citric acid-treated PG for cement load-bearing walls and road subgrade was improved to a certain extent [26,27,28]. The above results demonstrated the feasibility of secondary utilization of PG pretreated with citric acid. Whether the citric acid-treated PG is suitable to be utilized as an aggregate for cemented backfill to reverse the deterioration of the mechanical behavior of backfill and environmental pollution caused by fluoride is worthy of further study.

Herein, to study the influence of citric acid-treated PG on the properties of cemented backfill, the PG and backfill with and without pretreatment were prepared for comparative study. The effectiveness of the citric acid pretreatment on PG was verified by analyzing the total fluorine content in PG with and without pretreatment and the soluble fluoride concentration in PG washing solutions, as well as observing the microstructure evolution in scanning electron microscopy/energy dispersive X-ray spectroscopy (SEM/EDS). The effect of pretreated PG on the properties of the cementitious system was investigated through the backfill slurry and cemented backfill. Finally, the fluoride pollution caused by PG during the cemented backfill process was characterized by the concentration of soluble fluoride in the bleeding water of the backfill slurry and the toxic leaching solution from the hardened backfill. This work contributes to the application of pretreated PG in an in situ cemented backfill technique and provides theoretical support and relevance for eliminating environmental pollution caused by impure fluoride.

## 2. Materials and Methods

### 2.1. Raw Materials

PG and composite binder provided by Guizhou Kailin (Group) Co., Ltd., Guiyang, China, were used as raw materials in this study. The mixture proportion of the binder is *M* (yellow phosphorous slag): *M* (fly ash): *M* (cement clinker): *M* (lime) = 4:1:1:0.64–0.8. X-ray fluorescence (S4 Pioneer, Bruker, Fällanden, Switzerland) was utilized to determine the main chemical compositions of the PG and the binder, and the measurement results are listed in Table 1.

### 2.2. Batch Purification of PG

To explore the influence of citric acid pretreatment on PG, five different concentrations of citric acid solutions were considered in this study, ranging from 1% to 5%. The specific operation process was as follows: (1) PG was mixed with citric acid of different concentrations at a liquid-to-solid ratio of 2:1 and then stirred uniformly in an agitator at 200 r/min for 30 min. (2) The mixture was centrifuged to obtain the separated supernatant and PG. (3) The separated PG was washed with deionized water, stirring at 200 r/min for 10 min. (4) The mixture of step (3) was centrifuged again to obtain the separated supernatant and PG. Steps (3) and (4) were repeated to wash the PG with deionized water several times until the pH value of the separated supernatant was greater than 5. The supernatant separated from each wash was collected for subsequent analysis.

### 2.3. Test Methodology

**Figure 1:**
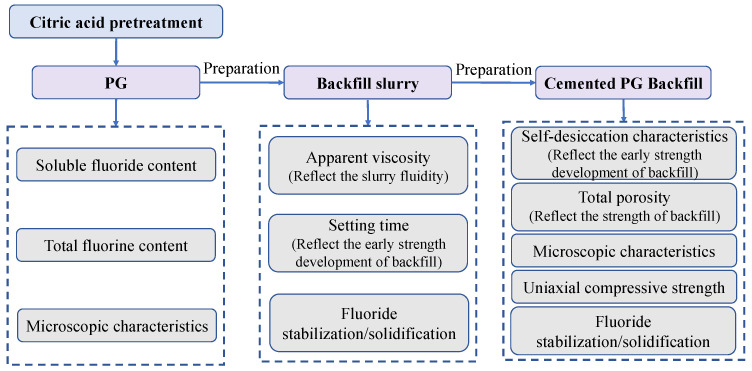
Flow diagram of the experiment.

In order to determine the optimization effect of citric acid pretreatment on the properties of materials involved in the whole cemented PG backfill process, including PG, backfill slurry and hardened backfill, a series of mechanical, physical and chemical tests were conducted, as illustrated in Figure 1. The details of the test procedure and method are introduced in the following sub-sections.

#### 2.3.1. Preparation of Cemented Backfill Samples

After washing PG with citric acid of different concentrations, 40% PG, 10% binder and 50% water were mixed in an agitator at a stirring speed of 200 r/min for 30 min to prepare the cemented PG backfill samples with a dimension of 40 mm × 40 mm × 40 mm. All backfill samples were cured in a curing chamber with a temperature of 20 °C and a humidity of 95%.

#### 2.3.2. Determination of Apparent Viscosity and Setting Time

The apparent viscosity of cemented backfill slurry was determined by DV-1 digital viscometer (Brookfield, WI, USA) in accordance with the American standard ASTM D2196-18 [29]. The initial setting time (IST) and final setting time (FST) of backfill slurry were determined using Vicat apparatus in accordance with the Chinese standard GB/T 1346-2011 [30].

#### 2.3.3. Suction Monitoring

Firstly, the prepared backfill slurry was poured into a plastic mold 15 cm in diameter and 20 cm in height. A matrix water potential sensor (MPS-6) with a measurement range of −9 to −100,000 kPa and an accuracy of ±0.1 kPa was then installed in the mold center, and the data were recorded in real time by connecting the EM60 data logger (Meter, San Francisco, CA, USA). The outside of the mold was wrapped with plastic wrap, leaving only a hole with a diameter of 2 mm at the mold bottom for draining excess water. Finally, the sensor device and the mold were placed in the curing chamber with the prepared backfill samples under the same conditions.

#### 2.3.4. Porosity Measurement

Nuclear magnetic resonance (NMR) spectroscopy is a reliable technique for measuring the pore structure of porous materials and has been widely used in many fields. In this study, the total porosity of cemented backfill was measured using the AniMR-150 NMR instrument (Niumag, Shanghai, China). After curing for 28 d and 90 d, the backfill samples were dry pumped for 6 h and wet pumped for 4 h in a vacuum saturator with a constant negative pressure of 0.1 MPa, and then immersed in water for another 48 h to ensure that the pores in backfills were filled with water. The calculation method of porosity was referred to in previous studies [31,32].

#### 2.3.5. Uniaxial Compressive Strength Test

After curing at various curing ages (7 d, 14 d, 28 d, 60 d and 90 d), the uniaxial compressive strength (UCS) of cemented backfill samples was measured by a WHY-200 (Hualong, Shanghai, China) hydraulic servo testing device in accordance with the Chinese standard JGJ/T 70-2009 [33]. Each trial was repeated three times and the average value was considered as the calculated UCS.

#### 2.3.6. Microstructure Test

After the UCS test, the central parts of the broken backfill samples were selected to be immersed in anhydrous ethanol to terminate the hydration reaction, and SEM and EDS examinations were performed by TESCAN-MIRA scanning electron microscope (Tescan, Bron, Southern Moravia, Czech Republic). The PG pretreated with and without citric acid was also tested.

#### 2.3.7. Toxicity Leaching Test

The fluoride concentration in cemented PG backfill samples was determined by a toxicity leaching test in accordance with the Chinese standard HJ 557-2010 [34]. The backfill samples were first sanded to a cube with a side length of less than 3 mm, and then put into a plastic container with deionized water at a liquid-solid ratio of 10:1 (L:kg). Subsequently, the container was placed on a vibrator and vibrated at an amplitude of 40 mm and a speed of 110 r/min for 8 h, then left to stabilize for 16 h. Finally, the supernatant was drawn from the container and filtered through a filter with a diameter of 0.45 μm for subsequent chemical measurements.

#### 2.3.8. Chemical Composition Measurement

The total fluorine content in PG was determined in accordance with the Chinese standard JC/T 2073-2011 [35]. First, 1 g PG sample (dry weight) and 10 mL deionized water were mixed and 12 mL HCl (1 + 1) was added. Then, the mixture was heated in a water-bath heating oven until it slightly boiled and was kept for 2 min. Finally, the leachate was filtered through a 0.45 μm filter and collected. The soluble fluoride content in backfill was measured in accordance with the Chinese standard HJ 557-2010. The concentration of soluble fluoride in the bleeding water of backfill slurry and the backfill leachate after the toxicity leaching test was determined by a fluorine ion-selective electrode (Mettler Toledo, Zürich, Switzerland), and the pH value was determined by Ohaus TSARTR 300 pH meter (Mettler Toledo, Zürich, Switzerland).

## 3. Test Results and Discussion

### 3.1. Removal of Fluoride from PG by Citric Acid Washing

#### 3.1.1. Soluble Fluoride Content in PG

**Figure 2:**
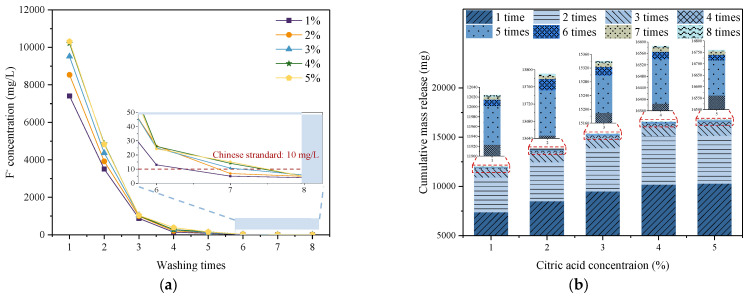
(**a**) Variation of F^−^ concentration in the supernatant with washing times and (**b**) the cumulative mass release of F^−^.

Figure 2 shows the change in soluble fluoride concentration and cumulative mass release in the supernatant as a function of washing times. In this test, PG was washed with citric acid solutions of varying concentrations in the first washing time. Deionized water was then used as the washing solution for the rest of the washing times to remove the fluoride and increase the pH of PG. The higher the F^−^ concentration detected in the supernatant, the more fluoride was washed out from the PG and the less fluoride remained in the PG.

Figure 2a depicts the variation of the F^−^ concentration in the supernatant with washing times. For five batches of PG, an F^−^ concentration of more than 7400 mg/L was detected in the supernatant after being washed with citric acid and centrifuged once. This is because the fluoride in PG can react with citric acid in the washing solution. On the one hand, citric acid can combine with soluble fluorides, such as NaF, to generate water-soluble Na_3_(C_6_H_5_O_7_)_2_ and HF. On the other hand, citric acid can also combine with insoluble fluoride to generate water-soluble HF, H_3_SiF_6_, H_3_FeF_6_ and H_3_AlF_6_ [36]. The reaction equations are as follows:

(1) $${2C}_{6}H_{8}O_{7}+3\mathrm{NaF}\to {\mathrm{Na}}_{3}{\left(C_{6}H_{5}O_{7}\right)}_{2}+3\mathrm{HF}$$

(2) $${4C}_{6}H_{8}O_{7}{+3\mathrm{Na}}_{2}{\mathrm{SiF}}_{6}\to {2\mathrm{Na}}_{3}{\left(C_{6}H_{5}O_{7}\right)}_{2}{+H}_{3}{\mathrm{SiF}}_{6}$$

(3) $${2C}_{6}H_{8}O_{7}{+2\mathrm{Na}}_{2}{\mathrm{AlF}}_{6}\to {2\mathrm{Na}}_{3}{\left(C_{6}H_{5}O_{7}\right)}_{2}{+H}_{3}{\mathrm{AlF}}_{6}$$

(4) $${2C}_{6}H_{8}O_{7}{+3\mathrm{Na}}_{2}{\mathrm{FeF}}_{6}\to {2\mathrm{Na}}_{3}{\left(C_{6}H_{5}O_{7}\right)}_{2}{+H}_{3}{\mathrm{FeF}}_{6}$$

(5) $${4C}_{6}H_{8}O_{7}{+3\mathrm{CaF}}_{2}\to {\mathrm{Ca}}_{3}{\left(C_{6}H_{5}O_{7}\right)}_{2}+6\mathrm{HF}$$

With the occurrence of the reaction, these fluorides are extracted from PG to the supernatant, which increases the F^−^ concentration in the supernatant. Moreover, the F^−^ concentration increased with the increasing citric acid concentration in the supernatant. In detail, with the increase in the citric acid concentration from 1% to 5%, the concentration of F^−^ rose from 7405 mg/L to 10,314 mg/L, which means that more fluorides will be washed out from PG when the citric acid concentration increases. This is because the solubility of fluoride, such as NaF, NaSiF_6_ and NaFeF_6_, enhances with increasing citric acid concentration [26]. Subsequently, the F^−^ concentration in the supernatant gradually decreased as the washing times increased. It was not until the washing times increased to 7–8 that the F^−^ concentration dropped to about 5 mg/L, which was lower than the national sewage discharge standard (10 mg/L). The above results indicate that the water-soluble fluorides generated by the reaction with citric acid can be easily removed from PG with a stream of water.

When the mass of PG is 500 g, the release mass of F^−^ in the cumulative 8 washes supernatant is depicted in Figure 2b. Obviously, the cumulative mass release of F^−^ in the first three washes was significantly higher, accounting for more than 96.5% of the accumulation of F^−^ in all washes. In subsequent washes, the mass release of F^−^ was significantly reduced. In addition, the concentration of citric acid was positively correlated with the mass release of F^−^. With the increasing citric acid concentration from 1% to 4%, the accumulation of F^−^ gradually increased from 12,024 mg to 16,586 mg by 38%. As the citric acid concentration rose to 5%, the increase in the cumulative mass release was relatively small, with only a 1.05% increment. This demonstrates that when the acid concentration increases to a certain value, further increasing the concentration of citric acid cannot wash out more fluoride, so it is recommended that the citric acid concentration for PG washing should not exceed 4%.

**Figure 3:**
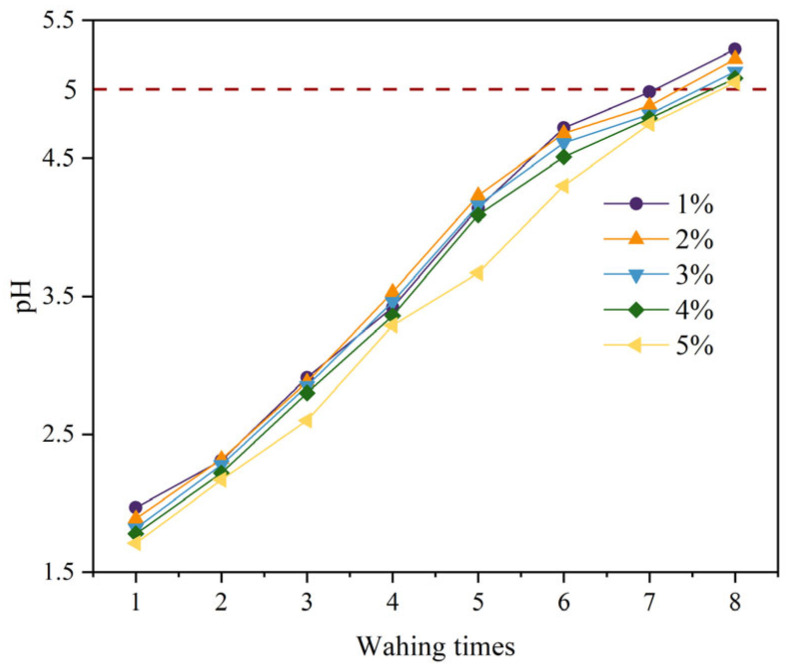
Variation of pH value of supernatant with washing times under different citric acid concentrations.

Figure 3 depicts the variation of the pH of the supernatant with washing times under different citric acid concentrations. In the first washing, the supernatant of the PG washed with citric acid had a very low pH of 1.71–1.97. This is because citric acid is an organic acid and the pH of citric acid with a concentration of 1–5% is 2.2–1.9. In the following washes, the pH of the supernatant gradually increased with increasing washing times, which was mainly due to two reasons. On the one hand, the residual acid adsorbed on the PG crystal surface is easily separated from the PG crystal surface and dissolved in washing solutions. On the other hand, soluble compounds such as HF produced by the reaction of citric acid and fluoride are also easily dissolved in water and can be removed through the stream of water. When washing 7–8 times, the pH value of the supernatant was greater than 5. Previous researchers have proved that when the pH of the supernatant is greater than 5, the hydration reaction will not be negatively affected [37]. It is therefore recommended that the PG should be washed 7–8 times to meet backfill requirements.

#### 3.1.2. Total Fluorine Content in PG

**Figure 4:**
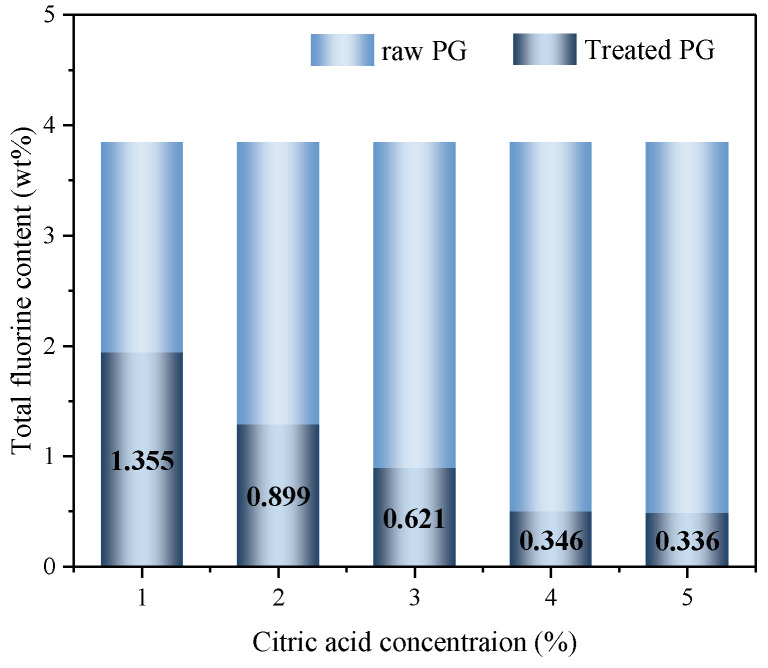
Total fluorine content in PG pretreated with different citric acid concentrations.

To further demonstrate that pretreated PG with citric acid can effectively remove fluoride, the value of total fluorine content in PG is illustrated in Figure 4. The total fluorine content in the raw PG was 3.845 wt%; when pretreated with citric acid at a concentration of 1%, the total fluorine content in PG dropped to 1.355 wt%, a decrease of 65%. When the citric acid concentration increased to 4%, the total fluorine content in PG decreased significantly to 0.346 wt%, with a decrease of 91% compared with raw PG. As the concentration of citric acid continued to increase, although the total fluorine content in PG still exhibited a downward trend, the decreasing amplitude was very small and tended to be gentle. However, the total fluorine content in PG with a citric acid concentration of 5% was only 0.01 wt% less than that with a citric acid concentration of 4%. Therefore, the increase in citric acid concentration from 4% to 5% has little effect on PG defluoridation, which is the same as the changing trend in Section 3.1.1. From the change in total fluorine content, it can also be inferred that the optimal concentration of citric acid to minimize the fluorine content in PG is 4%.

#### 3.1.3. Microscopic Characteristics of PG

**Figure 5:**
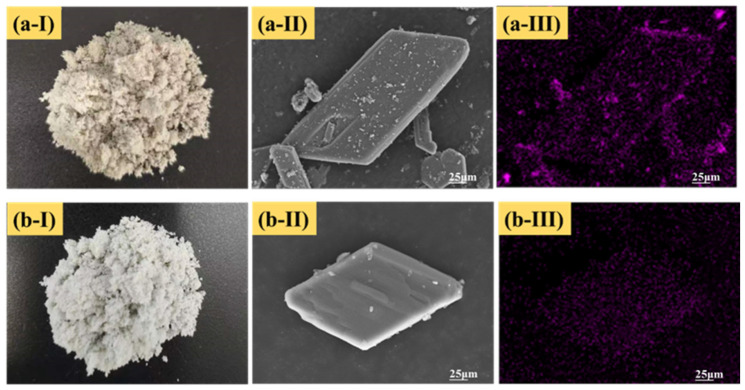
Appearance morphology, SEM image and EDS mapping for (**a-I–a-III**) raw PG and (**b-I–b-III**) PG pretreated with 2% citric acid.

Figure 5 illustrates the appearance morphology, SEM image and EDS mapping for raw PG and the PG pretreated with 2% citric acid. The appearance morphology shows that the color of the raw PG (Figure 5(a-Ⅰ)) was gray-black. After being pretreated with citric acid, it turned milky white (Figure 5(b-Ⅰ)). Previous research has shown that the whiter the color of PG, the lower the impurity content, which is an intuitive method to judge the purity of PG [38]. It can be seen from the SEM image that the massive number of tiny particles adhered to the surface of raw PG resulted in a rough crystal surface (Figure 5(a-Ⅱ)). In contrast, the crystal surface of pretreated PG was relatively smooth, with only several tiny particles attached (Figure 5(b-Ⅱ)). Despite repeated washing, the crystal morphology of the pretreated PG remained in a rhombic plate shape, and only the majority of tiny particles were removed from the PG crystal surface, indicating that citric acid washing would not damage the structure and shape of the PG crystal. Furthermore, EDS mapping detection showed that the element F distributions on the surface of PG samples with and without citric acid pretreatment were different. The F distribution on the surface of raw PG was relatively dense and extensive (Figure 5(a-Ⅲ)), while the distribution on the surface of pretreated PG was sparser (Figure 5(b-Ⅲ)) due to most of the F-bearing phases having been removed after treatment. This intuitively indicates that the citric acid washing had a good removal effect on impurities in PG.

### 3.2. Optimization of the Properties of Cemented Backfill Slurry

#### 3.2.1. Apparent Viscosity

**Figure 6:**
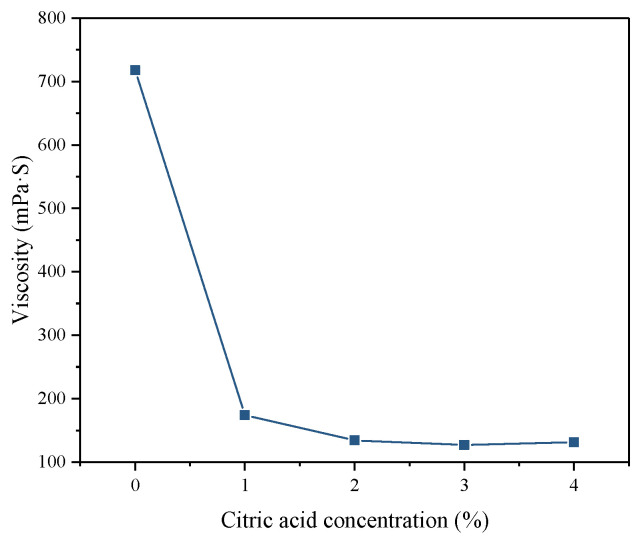
Viscosity of backfill slurry prepared by PG pretreated with different citric acid concentrations.

The viscosity of backfill slurry is a key parameter reflecting slurry fluidity. In practice, the lower viscosity reduces the resistance of pipeline transportation, thereby reducing the operating pressure of industrial pumps and cutting costs. Five batches of PG pretreated with different citric acid concentrations were used to make the backfill slurry and the slurry viscosity is depicted in Figure 6. The viscosity of the backfill slurry made by raw PG was 718 mPa·S, while the slurry viscosity of the treated PG reduced to 127–174 mPa·S. The higher viscosity of the backfill slurry made from raw PG is because the fluoride contained in the raw PG can react with the binder. Among them, soluble fluorides can react with Ca^2+^ from the binder to form Ca-F precipitations [39]. Insoluble fluorides, such as fluorosilicate, hydrolyze to F^−^ under the high pH condition brought about by the hydration reaction of the binder, and then combines with Ca^+^ to form Ca-F precipitations [40]. The reaction equations are as follows:

(6) $$F^{-}+{\mathrm{Ca}}^{2+}\to {\mathrm{CaF}}_{2}$$

(7) $${\mathrm{SiF}}_{6}^{2-}+{\mathrm{Ca}}^{2+}+{\mathrm{OH}}^{-}\to {\mathrm{CaF}}_{2}+{\mathrm{SiO}}_{2}+H_{2}O$$

The generated Ca-F precipitation adheres to the PG crystal surface, roughening the surface of PG and increasing the friction resistance between particles in the backfill slurry, which, in turn, shows a higher viscosity [41,42]. Additionally, the residual phosphate in PG can also react with the binder to generate Ca-P precipitations (such as Ca_3_(PO_4_)_2_ and Ca_5_(PO_4_)_3_), which are also adhered to the surface of PG crystals [23,43,44]. Notably, the contents of soluble and insoluble fluorides in PG were significantly reduced after citric acid washing, resulting in a decrease in viscosity of 75.77–82.31% compared with that of raw PG.

#### 3.2.2. Setting Time

**Figure 7:**
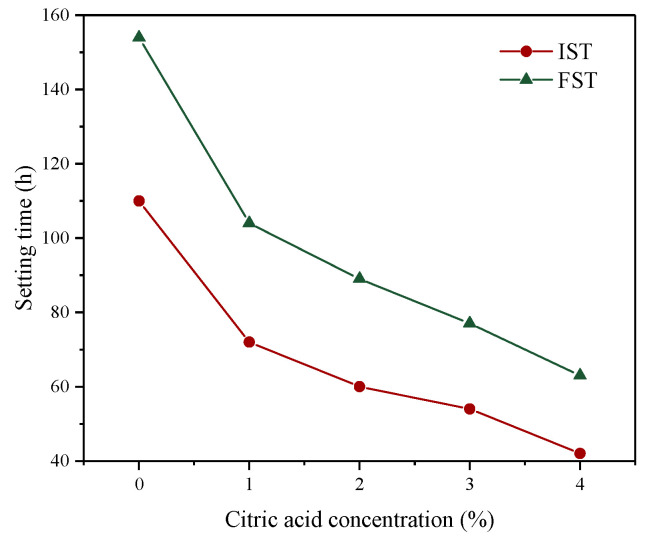
Setting time of backfill slurry prepared by PG pretreated with different citric acid concentrations.

The setting time of slurry is an important parameter affecting the early strength development of cemented backfill. The variation in the IST and FST of backfill slurry with different citric acid concentrations is depicted in Figure 7. The setting time of the backfill slurry prepared with raw PG was the longest, and the IST and FST were 110 h and 154 h, respectively. This is because the considerable content of fluoride contained in PG interferes with the hydration reaction. After adding the binder to PG, soluble and insoluble fluorides in PG immediately combine with Ca^2+^ from the binder to generate insoluble Ca-F precipitations, which leads to a reduction in the content of Ca^2+^. Moreover, Ca^2+^ is the main element for generating the hydration products (see Section 3.3.3), i.e., calcium silicate hydrate (C-S-H) gel and ettringite (AFt), resulting in a corresponding decrease in the content of hydration products [45]. Moreover, the fine crystals of Ca-F precipitation produced by the reaction are easily adsorbed on the surface of binder particles, lowering the dissolution rate of Ca^2+^ into the liquid and, thus, hindering further hydration of the binder [20]. The combination of these two factors causes the prolonged setting time. In addition, other impurities, such as phosphates, residual acids, organic matter, etc., can also affect the hydration process of cemented backfill, resulting in a slow solidification [40,46,47]. After citric acid pretreatment, the setting time of the backfill slurry was greatly reduced, which owed to a reduction in the impurities of PG by citric acid washing. With the increasing citric acid concentration from 1% to 4%, the IST and FST of backfill slurry decreased by 40–53% and 47–56%, respectively. A previous study has shown that, on the premise of ensuring transportation performance, the faster the setting time of slurry is, the better the early strength formation of backfill [48].

### 3.3. Optimization of the Mechanical Properties of the Cemented Backfill

#### 3.3.1. Self-Desiccation Characteristics

**Figure 8:**
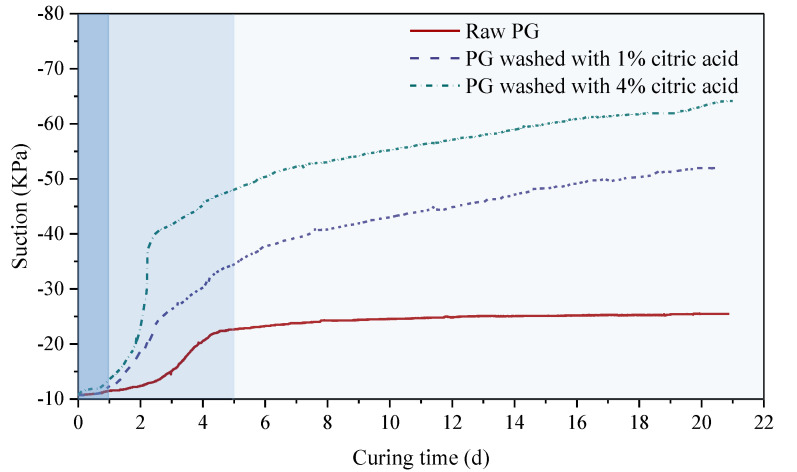
Suction development of backfill prepared by PG pretreated with different citric acid concentrations.

The self-desiccation of backfill is caused by water consumption during the hydration reaction, which decreases the excess pore-water pressure leading to suction development, thus accelerating the early strength development of backfill [49]. Figure 8 illustrates the influence of citric acid pretreatment on the suction behavior of cemented PG backfill cured for 21 d. Obviously, the suction value of the backfill was closely related to the curing time and citric acid concentration. In about 1 d, the suction curves of all backfills were at the platform stage and had no significant change with increasing curing time because the backfill slurry did not complete the initial setting within 1 d and was still in the slurry state containing a lot of free water.

With the increase in curing days, the suction curves of backfill entered the accelerated growth stage and the increase rate was obviously affected by the citric acid pretreatment. The backfill prepared by pretreated PG increased rapidly in about 1–3 d, while the untreated backfill increased rapidly in about 3–5 d. In this stage, the slurry gradually completed the initial and final setting, forming a hardened structure which consumed a lot of free water in the slurry, resulting in a rapid increase in its suction value. The rapid increase in suction value means that the hydration reaction process is accelerated, that is, the backfill pretreated with citric acid will generate strength earlier, which is conducive to mine backfill. With the further increase in curing days, the suction curves of the backfill prepared with raw PG transited to the platform stage again, while that of the pretreated backfill transited to the parabolic growth stage. During the 21 d of suction monitoring, the suction value of the backfill prepared with raw PG increased slowly from −10.77 KPa to −25.48 KPa, whereas that of the backfill pretreated with 1% and 4% citric acid increased by 41.19 KPa and 53.37 KPa, respectively. The above results illustrate that the suction value of the backfill prepared by pretreated PG is significantly greater than that of the untreated backfill, so citric acid washing has a significant optimization effect on the early strength development of the cemented PG backfill.

#### 3.3.2. Total Porosity

**Figure 9:**
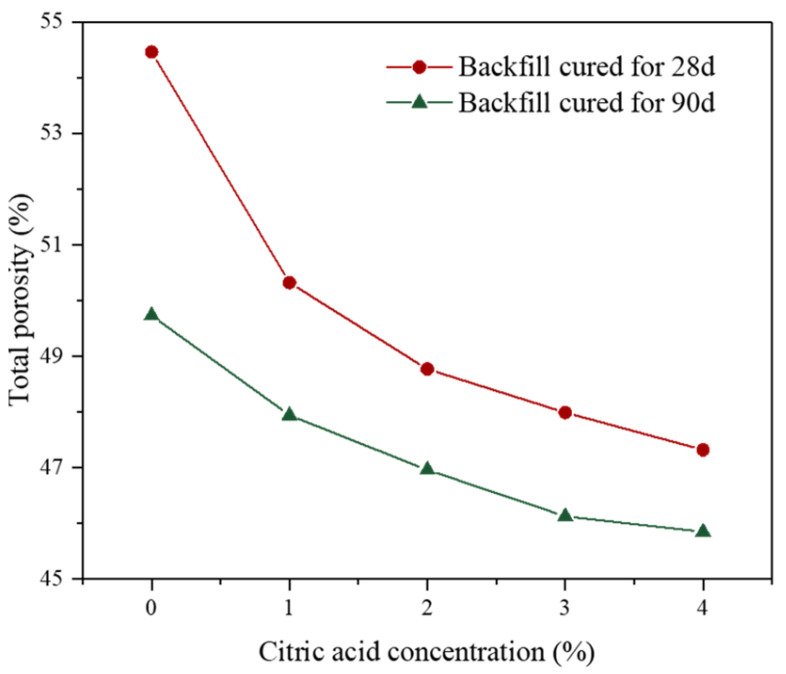
Porosity of backfill prepared by PG pretreated with different citric acid concentrations.

Recently, the impact of total porosity on the cemented backfill performance has received increasing attention [50]; it is found that a higher porosity usually corresponds to a lower backfill strength. Figure 9 shows the porosity results of the backfill cured for 28 d and 90 d. It can be seen that the porosity of all backfills decreased with the increment in curing time from 28 d to 90 d. This is because the cemented backfill is composed of aggregate PG and binder, so it can be considered as a continuous and random filler [51]. The pore volume between aggregates is filled by the hydration products of the binder, and the quantity of hydration products increases with increasing curing time, thereby reducing the backfill porosity. In addition, it was found that the porosity of the backfill prepared by pretreated PG was significantly lower than that of the untreated backfill. When the citric acid concentration was 1%, the porosity of the backfill after curing for 28 d was 50.32%, which was reduced by 4.14% compared with the raw PG. And, the porosity of the 90 d backfill was also significantly smaller than that of the untreated backfill. The result indicates that the hydration products of the backfill develop better after citric acid pretreatment, and the compactness of the backfill can be greatly improved. Moreover, with an increasing citric acid concentration from 1% to 4%, the porosity of the backfills after curing for 28 d and 90 d decreased from 50.32% to 47.32% and 47.94% to 45.85%, respectively, which implies that the internal hydration products of the backfill grew better by increasing the concentration of citric acid.

#### 3.3.3. Microstructure of Cemented PG Backfill

**Figure 10:**
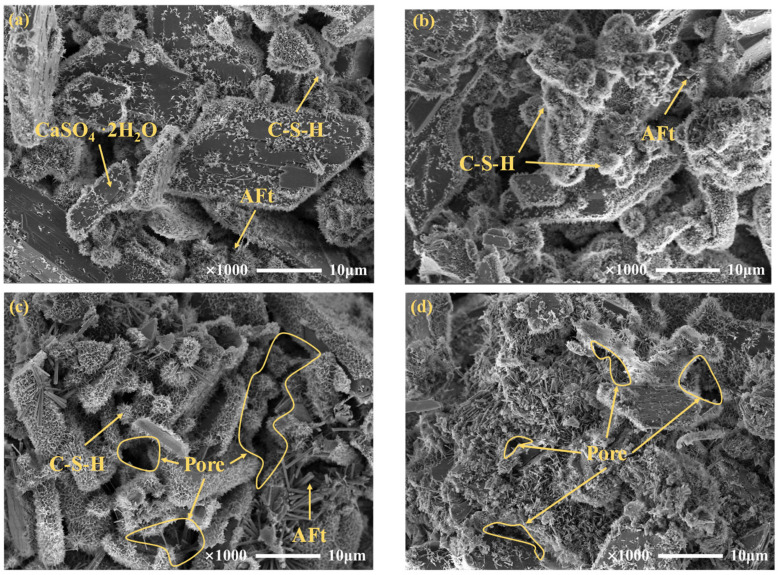
SEM images of the backfill: (**a**) cemented raw PG backfill cured for 28 d; (**b**) cemented pretreated PG backfill cured for 28 d; (**c**) cemented raw PG backfill cured for 90 d; (**d**) cemented pretreated PG backfill cured for 90 d.

To intuitively characterize the development of hydration products, SEM images with a magnification of 2000 times were selected to observe hydration products in the backfill prepared with raw PG and 4% citric acid pretreatment, as displayed in Figure 10. The hydration products of the backfill were mainly flocculent C-S-H gel and needle-like AFt. As shown in Figure 10a, after curing for 28 d, the plate-like PG crystals in backfill prepared with raw PG were still visible. Meanwhile, minor amounts of C-S-H gel and fine AFt attached to the surface of PG and interspersed in the pores, while the impurities adsorbed on the PG crystal surface could not be observed. Conversely, for the backfill prepared with pretreated PG and cured for 28 d (Figure 10b), plenty of hydration products were found and filled in pores of the backfill. It is well known that C-S-H can be filled to act as a cementation between aggregates, and AFt can interlock with C-S-H to make the structure denser. The combined effect of C-S-H and AFt can obviously enhance the backfill strength.

Figure 10c shows that compared with the backfill cured for 28 d, more C-S-H was generated in the 90 d backfill and the AFt grew stronger. However, there were still many pores between the hydration products and the microstructure was not dense. In contrast, in Figure 10d, the shape of C-S-H changed from flocculent to a more tight network structure, and the hydration products were well-filled between the PG crystals, making the pores tiny. It is concluded that citric acid pretreatment can ameliorate the microstructure of cemented backfill effectively by promoting the hydration reaction and reducing the backfill porosity.

#### 3.3.4. Strength Development

**Figure 11:**
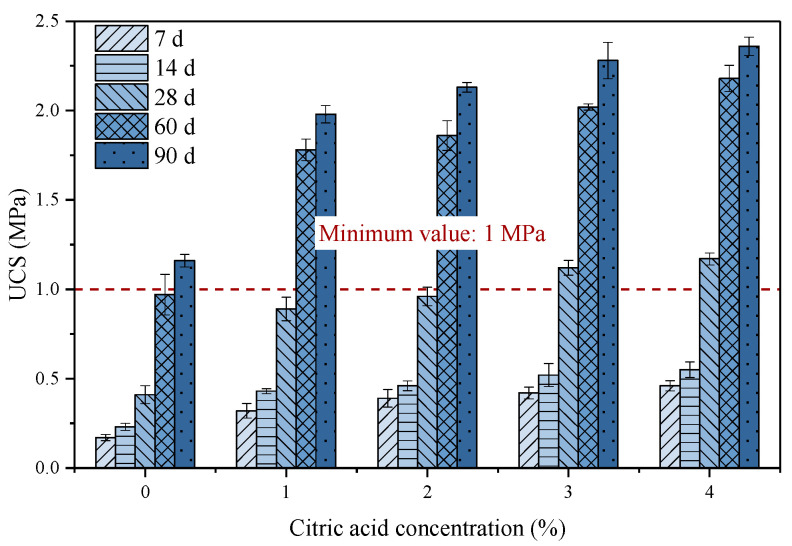
UCS of backfill prepared by PG pretreated with different citric acid concentrations.

UCS is one of the most vital indexes to evaluate the stability of goaf after backfill pumping. Figure 11 summarizes the UCS values of backfill samples after curing for different days. The UCS of backfill prepared with raw PG cured for 7 d was only 0.17 MPa, and the UCS value only increased to 0.41 MPa when the curing age increased to 28 d. The UCS of backfill pretreated with different citric acid concentrations cured for 7 d was 0.32–0.46 MPa, and the 28 d UCS climbed to 0.89–1.17 MPa. One possible reason for the restricted early strength development of the backfill prepared by raw PG is that the binder particles are covered by a film generated by the reaction of the impurities, such as soluble fluoride in PG with Ca^2+^, thus slowing down the early hydration process of the backfill. Although, as the hydration process proceeds, the film is broken and the process of early strength development of the fill slows down. Moreover, as depicted in Figure 11, the amount of hydration products in the cemented backfill prepared with raw PG is relatively few and does not form a well-lap with PG crystals. However, the PG pretreated with citric acid can weaken the negative effect of fluoride on hydration products, and thus improve the backfill strength.

Furthermore, as the concentration of citric acid reached 4%, the 90 d UCS of the pretreated backfill was 2.38 MPa, which was slightly higher than the UCS of 2.35 MPa for the backfill prepared by PG pretreated with 3% citric acid. As analyzed in Section 3.1, when the citric acid exceeds a certain concentration, few fluorides can be removed from PG, resulting in no significant improvement in strength, which is consistent with previous studies [26]. Furthermore, the UK EA provided a minimum value of 28 d UCS of 1 MPa in the S/S process [52,53]. Therefore, when the citric acid concentration was 3%, the UCS of 28 d backfill can reach the minimum request. The UCS value of all backfill increased in different degrees as the curing time continued to be extended with a similar growth trend as the 28 d backfill.

### 3.4. Optimization of Fluoride S/S during the Cemented Backfill Process

#### 3.4.1. Fluoride S/S in Backfill Slurry

**Figure 12:**
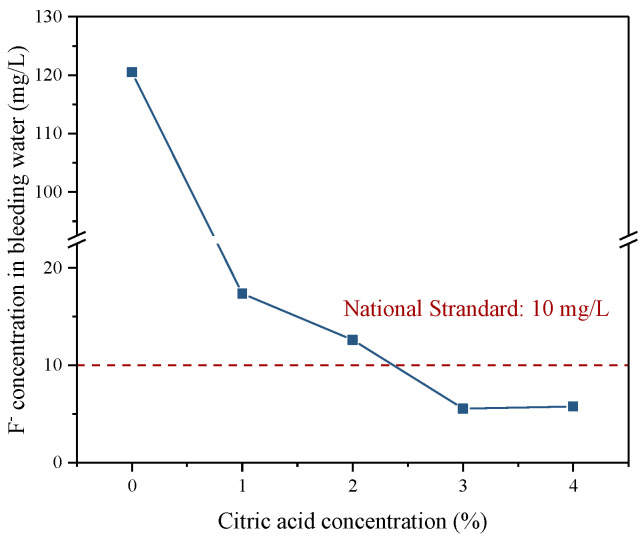
F^−^ concentration in the backfill slurry prepared by PG pretreated with different citric acid concentrations.

In this paper, special attention was paid to evaluating the mobility of the fluoride contained in the backfill with and without citric acid pretreatment. The concentration of F^−^ in the bleeding water of the backfill slurry was examined and the results are illustrated in Figure 12. Compared with an F^−^ concentration of 14,580 mg/L in raw PG, the backfill slurry made by the raw PG was 120.5 mg/L, indicating that the consolidation rate of fluoride in the mixing process of aggregate PG and binder is about 99.18%. The immobilization may be due to the substantial Ca^2+^ in the binder that can react with F^−^ in an alkaline environment to form Ca-F precipitation. Nevertheless, the F^−^ concentration of 120.5 mg/L in backfill slurry still far exceeded the national standard of 10 mg/L, which meant that some fluoride could not be immobilized. The high concentration of fluoride in slurry is likely attributed to two aspects. On the one hand, Ca^2+^ in the binder is incompletely utilized to immobilize the fluoride. PG contains not only fluoride but also other impurities, for example phosphate, which is prevalent in PG, which preferentially generate more insoluble precipitations than Ca-F compounds, resulting in insufficient content of Ca^2+^ to combine with the fluoride [39,43]. On the other hand, the solubility of the generated Ca-F precipitation is relatively high in an alkaline slurry, resulting in a concentration of fluoride hardly below 10 mg/L [16]. However, the concentration of F^−^ was significantly reduced in the bleeding water of the backfill slurry prepared by pretreated PG. When the citric acid concentration reached 3%, the measurable F^−^ concentration in the bleeding water was minimal at about 6 mg/L, which met the Chinese standard, indicating that the environmental impact of the pretreated PG on the backfill slurry was within the acceptable range.

#### 3.4.2. Fluoride S/S in Backfill

**Figure 13:**
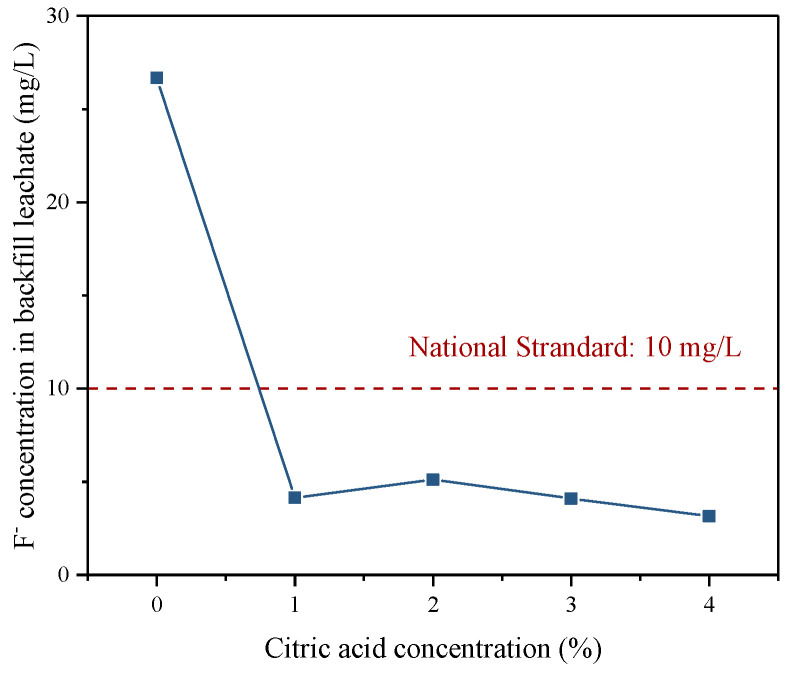
F^−^ concentration in the leachate of backfill prepared by PG pretreated with different citric acid concentrations.

In the leaching solution of the backfill cured for 28 d, the F^−^ concentration of all samples was lower than that of the bleeding water in the backfill slurry (see Figure 13), which might be related to the continuous hydration of the binder, providing high physical adsorption [54]. Regardless of the fluoride content in the pretreated PG, the concentration of F^−^ in the backfill leachate was always below the standard limits. In comparison, the backfill prepared with raw PG generated fewer hydration products than the backfill prepared by the pretreated PG and had a correspondingly weaker S/S capacity, with 17 mg/L of F^−^ concentration in the backfill leachate. Fluoride in the backfill prepared by PG pretreated with citric acid could be well immobilized since the pretreated PG has been purified with a relatively low fluoride content.

## 4. Conclusions

The high fluorine content of PG is a complex issue affecting the effectiveness of the in situ cemented PG backfill. In this study, the PG was first washed with citric acid to explore the optimization of the pretreated PG on the physico-mechanical properties of the backfill and assess the environmental behavior of the fluoride during the cemented PG backfill process. The following conclusions can be drawn:

(1) The content of fluoride washed out from PG increased with increasing citric acid concentrations and decreased with increasing washing times. Considering the efficiency of defluorination, the optimal citric acid concentration and washing times were determined to be 4% and 7–8 times, respectively.
(2) After citric acid pretreatment, the viscosity and setting time of the backfill slurry were greatly reduced due to the reduction in fluoride in pretreated PG. Therefore, the citric acid washing pretreatment is beneficial to the pipeline transportation of backfill slurry and the early strength development of hardened backfill.
(3) The citric acid pretreatment effectively improved the microstructure of cemented PG backfill by promoting the hydration reaction and reducing the backfill porosity, thus significantly enhancing the backfill strength. Furthermore, the use of pretreated PG as an aggregate could greatly reduce the usage and cost of the binder in actual production.
(4) During the cemented PG backfill process, the hydration reaction had a good S/S effect on the fluoride in the PG. Remarkably, when the PG was pretreated with a citric acid concentration of more than 3%, the concentration of fluoride in bleeding water and the backfill leachate was below the national standard limit, which is conducive to reducing the surrounding environmental pollution.

## Figures and Tables

**Table 1:** Chemical compositions of PG and composite binder.

Chemical Composition	PG (wt%)	Binder (wt%)
SO_3_	54.93	5.72
CaO	36.99	47.94
P_2_O_5_	2.03	1.56
SiO_2_	1.90	26.01
Al_2_O_3_	0.56	8.25
Fe_2_O_3_	0.44	4.14
K_2_O	0.14	1.04
MgO	0.11	1.41
Na_2_O	0.07	0.54
TiO_2_	0.05	0.63
F	1.38	-

## Data Availability

The data presented in this study are available on request from the corresponding author.
